# Neighborhood Retail Food Environment, Diet Quality and Type 2 Diabetes Incidence in 4 Dutch Cohorts

**DOI:** 10.1016/j.tjnut.2025.04.022

**Published:** 2025-04-30

**Authors:** Nicolette R den Braver (Nicole), Jeroen Lakerveld, Femke Rutters, Brenda WJH Penninx, Ellen Generaal, Marjolein Visser, Erik J Timmermans, Jeroen HPM van der Velde, Frits R Rosendaal, Renee de Mutsert, Esther Winters-van Eekelen, Johannes Brug, Joline WJ Beulens

**Affiliations:** 1 Department of Epidemiology and Data Science, Amsterdam University Medical Centers, VU University Medical Center, Amsterdam, the Netherlands; 2 Health Behaviour and Chronic Disease, Amsterdam Public Health Research Institute, Amsterdam, the Netherlands; 3 Upstream Team, Amsterdam University Medical Centers, Vrije Universiteit Amsterdam, Amsterdam, the Netherlands; 4 Department of Psychiatry, Amsterdam University Medical Centers, VU University Medical Center, Amsterdam Public Health Research Institute, GGZ inGeest, Amsterdam, the Netherlands; 5 Department of Infectious Diseases, Public Health Service of Amsterdam, Amsterdam, the Netherlands; 6 Infectious Diseases, Amsterdam Institute for Immunology and Infectious Diseases, Amsterdam, the Netherlands; 7 Global Health, Amsterdam Public Health Research Institute, Amsterdam, the Netherlands; 8 Department of Health Science, Vrije Universiteit, Amsterdam Public Health Research Institute, Amsterdam, the Netherlands; 9 Julius Center for Health Sciences and Primary Care, University Medical Center Utrecht, Utrecht University, Utrecht, the Netherlands; 10 Department of Clinical Epidemiology, Leiden University Medical Center, Leiden, the Netherlands

**Keywords:** food environment, type 2 diabetes, exposome, dietary pattern, diet quality

## Abstract

**Background:**

Current evidence on the associations between the food environment and type 2 diabetes (T2D) is inconsistent and did not investigate the behavioral mediating pathway.

**Objectives:**

To investigate whether accessibility of food retailers in the residential neighborhood is associated with T2D incidence in 4 Dutch prospective cohorts, and whether this is mediated by diet quality.

**Methods:**

In this prospective multicohort study, we included 4 Dutch cohort studies (n_total_ = 10,249). Nearest distances from all participants’ home to supermarkets, fast-food outlets, and green grocers were calculated at baseline (2004–2012). Incidence of T2D during follow-up was assessed with cohort-specific measures. T2D incidence ratios (IRs) adjusted for demographics, lifestyle, and environmental factors were estimated using Poisson regression in each cohort, and results were pooled across cohorts using a random-effects model. In 2 cohorts (*n* = 7549), mediation by adherence to the Dutch Healthy Diet index 2015 (DHD15-index; range, 0–13) was investigated using linear and Poisson regression analyses.

**Results:**

Over a mean follow-up of 7.5 y, 569 (5.6%) participants developed T2D. Mean(standard deviation [SD]) age in the cohorts ranged from 41.1(12.9) to 67.4(6.8) y. No associations were observed between accessibility of different food retailers and T2D incidence: β_supermarket_, 0.02 (−0.01, 0.06); β_fast-food_, −0.01(−0.04, 0.03); β_green grocer_, 0.01(−0.05, 0.07). Mediation analyses indicated that every 100 m living further from a supermarket or green grocer was associated with lower adherence to DHD15: β_supermarket_ = −0.1 (95% confidence interval [CI]: −0.3, 0.0) and β_green grocer_ = −0.1 (95% CI: −0.1, 0.0), whereas living further away from fast-food associated with higher adherence (β_fast-food_ = 0.1 [95% CI: 0.0, 0.2]). Higher adherence to DHD15 was associated with lower T2D incidence (IR = 0.93 [95% CI: 0.88, 0.99]).

**Conclusions:**

Spatial accessibility of food retailers was not associated with risk of T2D. Nevertheless, consistent associations in hypothesized pathways were observed, such that spatial accessibility to healthier food retailers was associated with higher diet quality and spatial accessibility of unhealthier retailers with lower diet quality. Higher diet quality, in turn, was associated with lower T2D risk.

## Introduction

Type 2 diabetes (T2D) is a burdensome chronic disease, with 537 million cases worldwide in 2021 and 783 million estimated cases by 2045 [1]. Unhealthy dietary behaviors pose an important risk factor for T2D [2,3]. Consequently, the obesogenic food environment has gained increasing attention as a potential upstream determinant of unhealthy diet and T2D risk [[4], [5], [6]]. Neighborhood characteristics associated with dietary behaviors have been widely studied. These studies focused mostly on the retail food environment (RFE). Two systematic reviews [5,7] and 2 narrative reviews [8,9] were published on this topic, concluding that results for the association between RFE and T2D were inconsistent.

Although substantial evidence is available on the intermediate pathway between diet and T2D [10,11], the connection between the food environment and diet remains underexplored, and the comparability between studies is generally weak [4,5]. For instance, measures of exposure to the residential food environment vary considerably across studies. Studies using subjective measurements of RFE indicated that higher perceived availability of healthy RFE is associated with a healthier diet and lower risk of T2D or prevalence of T2D. However, studies that used objective measures of food environment (both densities and distances) or a relative ratio measure of healthy compared with unhealthy outlets (involving an arbitrary choice to what is healthy or unhealthy) showed mixed results [4,5]. Although evidence for objectively assessed RFE is important, e.g., to make policy recommendations, the methods used so far are inconsistent.

Moreover, studies that used objective measures of the food environment were often hampered by insufficient heterogeneity in the study area and a cross-sectional study design. In the Netherlands, a nationwide cross-sectional study indicated that the presence of a fast-food outlet within a small buffer around the home (100–500 m) was associated with a slightly higher T2D prevalence in urban areas [12]. Another study that covered 3 rural provinces in the Netherlands matched these conclusions with BMI as outcome, however, again with a cross-sectional design [13]. Furthermore, international evidence suggests that cross-sectional and prospective designs produced different associations [14].

In general, current prospective studies observed no associations for objectively measured densities of fruit and vegetable markets [15], fast-food outlets [16], or ratios of retail food environments with T2D incidence [[16], [17], [18]]. Of the 4 prospective studies that investigated fast-food exposure specifically, 3 indicated that a relatively high access to fast-food outlets—compared with total food outlets—was associated with a higher incidence of T2D [[19], [20], [21]]. However, few studies investigated the food environment comprehensively (including several retailer types) in the same study and none investigated potential mediating pathways through dietary intake.

Hypothetically, exposure to unhealthier food environments would make people consume more unhealthier foods, which in turn may lead to higher T2D incidence. Diabetes is a distal endpoint from exposure to the food environment, whereas many studies have relied on the hypothesis that diet quality mediates the association between food environment and T2D [17, [22], [23], [24]].

Therefore, in this prospective study, we aimed to investigate whether accessibility of food retailers in the residential neighborhood is associated with T2D incidence across 4 Dutch prospective cohort studies, and whether this association is mediated by diet quality. We have pooled the results from 4 prospective cohort studies, using the same methods across all cohorts, to ensure comparability across these studies.

## Methods

### Study setting

The present study included 4 Dutch prospective cohorts, enriched with exposure information via the Geoscience and Health Cohort Consortium (GECCO) from a variety of regions in the Netherlands [25]. Cohorts with a baseline measurement after 2004, a 6-digit residential postcode (PC6), and a baseline and follow-up measurement of T2D were included. Included cohorts were the second wave of the Hoorn Study (HS) [26], the Longitudinal Study of Aging (LASA) [27,28], the Netherlands Study of Depression and Anxiety (NESDA) [29], and the Netherlands Epidemiology of Obesity (NEO) Study [30]. Cohort characteristics are summarized in Table 1 [[27], [28], [29], [30]] and described in more detail in Supplemental Materials. Study participants with prevalent T2D at baseline and those with missing information on outcome or exposure were excluded from our analyses.

**TABLE 1:** Cohort characteristics.

Cohort	Baseline	Maximum follow-up duration (y)	Study area	T2D assessment	Dietary assessment	Additional info
HS [26]	2006–2007	7	Hoorn	Fasting blood glucose, HbA1c	FFQ at baseline	
LASA [27,28]	2005–2006	10	Greater area of Amsterdam, Zwolle, Oss	GP diagnosis, medication use, self-report	—	Older adults
NEO [30]	2008–2012	10	Greater area of Leiden and Leider Dorp	GP diagnosis or medication use from medical record	FFQ at baseline	Oversampling of people with BMI of ≥ 27 kg/m^2^
NESDA [29]	2004–2007	6	Amsterdam, Leiden, Groningen	Fasting blood glucose, self-report, medication use	-	Recruited from general population, primary care and mental health care. Over-representation of persons with depression/anxiety.

*Abbreviations:* BMI, body mass index; FFQ, Food Frequency Questionnaire; GP, general practitioner; HBA1c, glycated hemoglobin; HS, Hoorn Studies; LASA, Longitudinal Study of Aging; NEO, the Netherlands Epidemiology of Obesity; NESDA, the Netherlands Study of Depression and Anxiety; T2D, type 2 diabetes.

### Exposure—RFE

The RFE data were obtained from the Dutch company Locatus, which performs regular field audits from 2004 onward, including all retailers in the Netherlands. Through these field audits, information on the type of food retailer and x- and y-coordinates of food retailer locations was obtained. Locatus categorized branches of retailers that we further categorized into the following 3 categories: supermarkets, grocery stores, and fast-food outlets, as shown in Table 2. The validity of Locatus data was tested against a field audit in selected areas across the Netherlands in 2019. This validation study showed that the location and classification of grocery stores (e.g., supermarkets, green grocers) and food outlets (e.g., restaurants, fast-food restaurants) were “good” to “excellent.” For instance, the positive predictive value for location of all food retailers was 0.90, concordance was 0.83, and Kappa was 0.58 [31]. Participants’ residential PC6 centroids were transformed to x- and y-coordinates. Euclidean (i.e., circular) distance to nearest food retailers was calculated as the distance from these residential coordinates to the closest supermarket, green grocer, and fast-food outlet. We chose these 3 outlets because we expected the clearest associations with the outcomes. These distances were used as a continuous variable (per 100 m), as well as tertiles for further analyses. For our sensitivity analyses, counts of supermarkets, green grocers, or fast-food outlets were also calculated within a 400-m Euclidean buffer around the PC6 centroid, as a measure of the presence of these types of retailers.

**TABLE 2:** Categorization of food retailers according to Locatus branches and definitions, into categories of food retailers used for analyses.

Food retailer category for analyses	Locatus branches composing the main category	Definition of branches
Supermarket	Supermarket	Store selling a wide range of food and nonfood products which are used on a daily basis. Floor area should at least be 150 m^2^.
Green grocers	Vegetable/fruit	Main provision of potatoes, vegetables, and fruit.
Fast-food outlets	Fast-food	Main provision of mostly deep-fried products that are ready for consumption in few minutes after ordering. Usually there is no table service available.
	Delivery/takeaway	Main provision of meals that are not consumed in the store, but are collected or delivered.
	Grillroom/shawarma	Main provision of grill products such as shawarma, kebab etc.

### Outcome—T2D assessment

Assessment of T2D was performed with cohort-specific methods. Some cohorts obtained the incidence of T2D through screening at study visits and therefore had no exact time-to-event data. Participants were classified as having T2D if either of the following criteria was fulfilled, depending on data availability in the cohort: fasting plasma glucose levels ≥7.0 mmol/L, HbA1c levels ≥ 6.5%, 2-h glucose after oral glucose tolerance test ≥ 11.1 mmol/L, diabetes medication use (ATC code A10), diagnosis by a general practitioner, and/or self-reported T2D (Table 1).

### Mediator—diet quality

In the HS and NEO cohorts, participants self-reported dietary intake at baseline, using the same validated Food Frequency Questionnaire (FFQ), which was used for the mediation analyses [32,33]. The FFQ data were used to construct the Dutch Healthy Diet index 2015 (DHD15-index) as a measure of diet quality. Details of the index and its association with T2D incidence are described in detail elsewhere [10,34]. In short, the index includes *1)* adequacy components, of which a higher intake is desirable, e.g., vegetables, fruits, legumes, tea; *2)* moderation components of which intake should be reduced, e.g., red/processed meat, sodium, sugar sweetened beverages; *3)* optimum components of which an optimal intake range is advised, i.e., dairy; *4)* ratio components of which replacement of products is advised, e.g., whole-grain/refined grain ratio, liquid/solid fat ratio; and *5)* quality component where a specific product is advised, i.e., filtered coffee, rather than unfiltered. Each component was assigned a score between 0 and 10, where zero was low adherence and 10 indicated high adherence to the dietary guidelines. The FFQ used in this study did not provide information on added salt and type of coffee, and therefore these food groups were excluded from the index, resulting in a DHD15-index between 0 (low adherence) and 130 (high adherence) (Supplemental Table 1), we adjust this score to range between 0 and 13 for analytical purposes.

### Covariates

Questionnaires on demographics and lifestyle behaviors were administered in all cohorts. We categorized educational attainment according to the definition of the Statistics Netherlands (CBS) into low (primary school, no education), middle (secondary education, lower/secondary vocational education), and high (higher vocational education, university, doctoral degree) for all cohorts. We used education as a proxy for SES because this variable was suitable for harmonization over the cohorts, and earlier research showed it to be a strong predictor of socioeconomic position [35]. Ethnicity was reported as a percentage of Caucasians as opposed to other ethnicities. Only for NESDA and NEO ethnicity was used as a covariate, because HS and LASA consisted of 99%–100% Caucasian population. Smoking was categorized as never, former, and current smoker. Physical activity was self-reported through a short questionnaire to assess health-enhancing physical activity (SQUASH) [36] in NEO and HS, the LASA Physical Activity Questionnaire (LAPAQ) in LASA [37] and the short-form International Physical Activity Questionnaire (IPAQ) [38] in NESDA. From these questionnaires, we assessed total physical activity (PA) (e.g., walking, playing sports, domestic work, etc.) as hours per week (irrespective of PA intensity). Weight and height were measured in all studies. Degree of urbanization was categorized into 5 categories by CBS, ranging from not urbanized (<500 addresses per km^2^) to extremely urbanized areas (>2500 addresses per km^2^). Because of cohort-specific sampling methods, NESDA analyses were additionally adjusted for episodes of clinical depression in lifetime (assessed using the Composite International Diagnostic Interview), and LASA analyses were additionally adjusted for cohort (cohort 1 and cohort 2).

### Statistical analyses

Participants with missing data on residential address, T2D at follow-up, and prevalent T2D at baseline were excluded (HS: *n* = 85; LASA: *n* = 272; NESDA: *n* = 1180; NEO: *n* = 726). Missing values in covariates (not mediators) were imputed if missing was >5%. Multiple imputation was performed using 10 imputed datasets, with multiple mean matching, and pooled according to Rubin’s rules. T2D incidence ratios (IRs) were obtained for the association of distance to *1)* supermarkets; *2)* green grocers; and *3)* fast-food outlets with T2D incidence through Poisson regression analyses with robust standard errors, due to potentially high incidence of the outcome. Since not all cohorts had time-to-event data, we used a Poisson regression approach adjusting for follow-up duration. Distance was investigated as cohort-specific tertiles (T) to supermarkets, grocery stores and fast-food outlets, separately, and for continuous distance (per 100 m). Three adjustment models were computed per food retailer: model 1 was adjusted for age, sex, follow-up duration, ethnicity (in NESDA and NEO only); model 2 was additionally adjusted for education, physical activity, smoking and cohort-specific confounders (depression in NESDA, cohort in LASA); model 3 was additionally adjusted for the distances to the other 2 food retailer types, because food retailers may be clustered.

**FIGURE 1:**
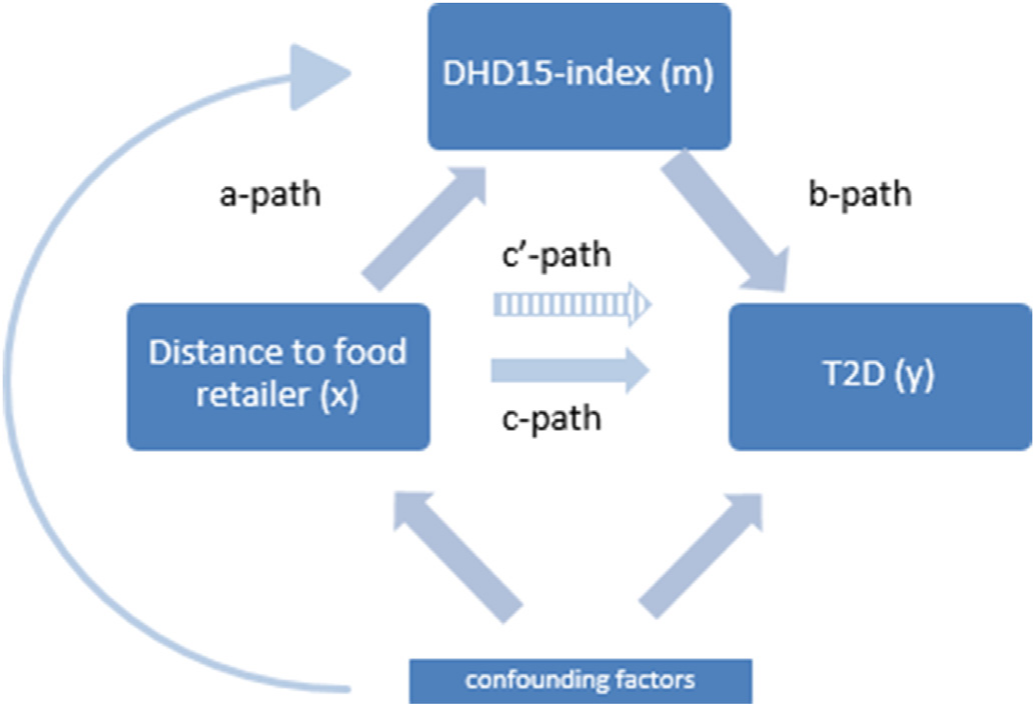
Mediation model of the association between distance to food retailers and type 2 diabetes (T2D) incidence, mediated by adherence to the Dutch Healthy Diet Index (DHD15-index). Path c represents the main association (x–y), and path c’ represent the main association (x–y), additionally adjusted for the mediator (m). Path a represents the association between distance to the food retailer (x) and adherence to the DHD15-index (m). Path b represents the association between adherence to the DHD15-index (m) and T2D incidence (y). DHD15-index, Dutch Healthy Diet Index; T2D, type 2 diabetes.

Mediation by diet quality was investigated using mediation analyses in the fully adjusted model, and analyses were performed for exposure to supermarkets, grocery stores, and fast-food outlets separately. In contrast to Baron & Kenny’s causal steps framework, we also investigated mediation if the main associations were nonsignificant, as it can offer insight into why associations are observed or not (i.e., contradicting associations, or significance only in parts of the association) [39]. We calculated proportion mediated (a∗b)/(a∗b+c’) only if there was significant mediation, the total effect (c-path) was in the same direction as the indirect path (a∗b) and whether the indirect effect was smaller than the total effect. With linear regression the a-path (distance to food outlets—DHD15-index) was estimated and the b-path (DHD15-index—T2D) and c’-path (continuous distance—T2D, adjusted for DHD15-index) were estimated (Figure 1) with Poisson regression. The indirect effect through the mediator was calculated with the product of coefficients method (a∗b) [40].

Effect modification was tested for age, sex, education, and urbanization level, in the association analyses with continuous distance (per 100 m). If consistent effect modification was observed across >2 cohorts, the pooled analyses were stratified for the effect modifier. If effect modification was not consistent, stratified analyses for those cohorts where effect modification was observed are presented in the Supplemental Materials.

Analyses were separately performed for each cohort, and effect estimates were then pooled over the cohorts using a random-effects model, from which pooled incidence ratios were presented as well as I^2^ statistic for heterogeneity over the cohorts. Analyses were performed in STATA version 14.

### Sensitivity analyses

For our sensitivity analyses, we used multiple definitions of exposure to the RFE, such as distances and densities, as suggested previously [41]. As 2 recent studies observed associations between presence of fast-food and BMI in smaller buffers (100–500 m) [12,13], and because the mean distance to the most abundant outlets (supermarkets and fast-food retailers) in our cohorts varied between 321 and 516 m, we chose to use a 400-m Euclidian buffer. We used the presence (i.e., 0 or >0 outlets) of supermarkets, fast-food outlets, and green grocers as the main determinant in our sensitivity analyses. We additionally assessed the associations between distance to food retailers and DHD15 scores for each food group, to gain insight whether any specific food groups were driving associations.

## Results

### Baseline characteristics

Mean (SD) age of the cohort participants ranged from 41.1 (12.9) y in NESDA to 67.4 (6.8) y in LASA. The cohorts consisted of 53% women in NEO to 67% in NESDA. Highest percentage of current smokers was observed in NESDA (32%) and lowest in LASA (15%). There was a variety in urban and rural regions over the cohorts, where NESDA had highest percentages of extremely urbanized areas (36%), and LASA had most rural areas (39%). Characteristics are summarized by cohort in Table 3.

**TABLE 3:** Baseline characteristics of study participants, stratified by cohort, and presented as *n*(%), mean (SD), or median [IQR].

	HS	LASA	NESDA	NEO
	*N = 1656*	*N = 899*	*N = 1801*	*N = 5893*
Sex (% male)	776 (46.9)	395 (43.9)	554 (32.8)	2754 (46.7)
Age (y)	53.5 (6.5)	67.4 (6.8)	41.1 (12.9)	55.6 (6.0)
Education (% high)	633 (39.1)	214 (23.8)	710 (39.4)	1776 (30.4)
Total physical activity (h/wk)	21.1 (15.0)	17.0 (12.7)	22.0 (14.1)	36.6 (19.4)
Smoking (current)	293 (17.8)	133 (14.8)	573 (31.8)	951 (16.1)
Ethnicity (% Caucasian)	(±100%)	895 (99.4)	1676 (93.1)	5602 (95.2)
BMI (kg/m^2^)	25.9 (3.7)	27.2 (3.9)	25.1 (4.7)	29.8 (4.7)
DHD15-index	65.6 (±14.3)	NA	NA	68.5 (±14.5)
**Environmental characteristics**				
Municipalities (*n*)	1	71	300	84
**Degree of urbanization**				
Extremely urbanized	0	157 (17.6)	645 (35.8)	1025 (17.7)
Strongly urbanized	697 (42.1)	181 (20.1)	302 (16.8)	1550 (26.7)
Moderately urbanized	430 (26.0)	61 (6.8)	287 (15.9)	1667 (28.7)
Hardly urbanized	354 (21.4)	144 (16.0)	162 (9.0)	546 (9.4)
Not urbanized	175 (10.6)	348 (38.7)	362 (20.1)	1014 (17.5)
**Distances (m)**				
**Supermarket**	476.7 (226.4)	428.9 [483.4]	391.4 [364.2]	465.0 (306.4)
**Fast-food outlets**	516.9 (466.7)	396.9 [521.3]	321.2 [425.4]	480.9 (367.1)
**Green grocers**	912.7 (491.6)	1239.9 [928.6]	636.1 [806.0]	818.2 (606.6)
**Presence within 400 m (% yes)**				
**Supermarket**	666 (40.2)	405 (45.1)	902 (50.1)	2763 (46.9)
**Fast-food outlets**	603 (36.4)	439 (48.9)	1057 (58.7)	3017 (51.2)
**Green grocers**	260 (15.7)	235 (26.1)	528 (29.3)	1393 (23.6)

*Abbreviations:* Abbreviations: BMI, body mass index; DHD15-index, Dutch Healthy Diet 2015 index; HS, Hoorn Studies; IQR, interquartile range; LASA, Longitudinal Study of Aging; NEO, the Netherlands Epidemiology of Obesity; NESDA, the Netherlands Study of Depression and Anxiety; SD, standard deviation; T2D, type 2 diabetes.

Degree of urbanization categories: not urbanized: <500 address per km^2^; hardly urbanized: 500–1000 addresses per km^2^; moderately urbanized: 1000–1500 addresses per km^2^; strongly urbanized: 1500–2500 addresses per km^2^; and highly urbanized: 1000–1500 addresses per km^2^.

### Pooled analyses

**FIGURE 2:**
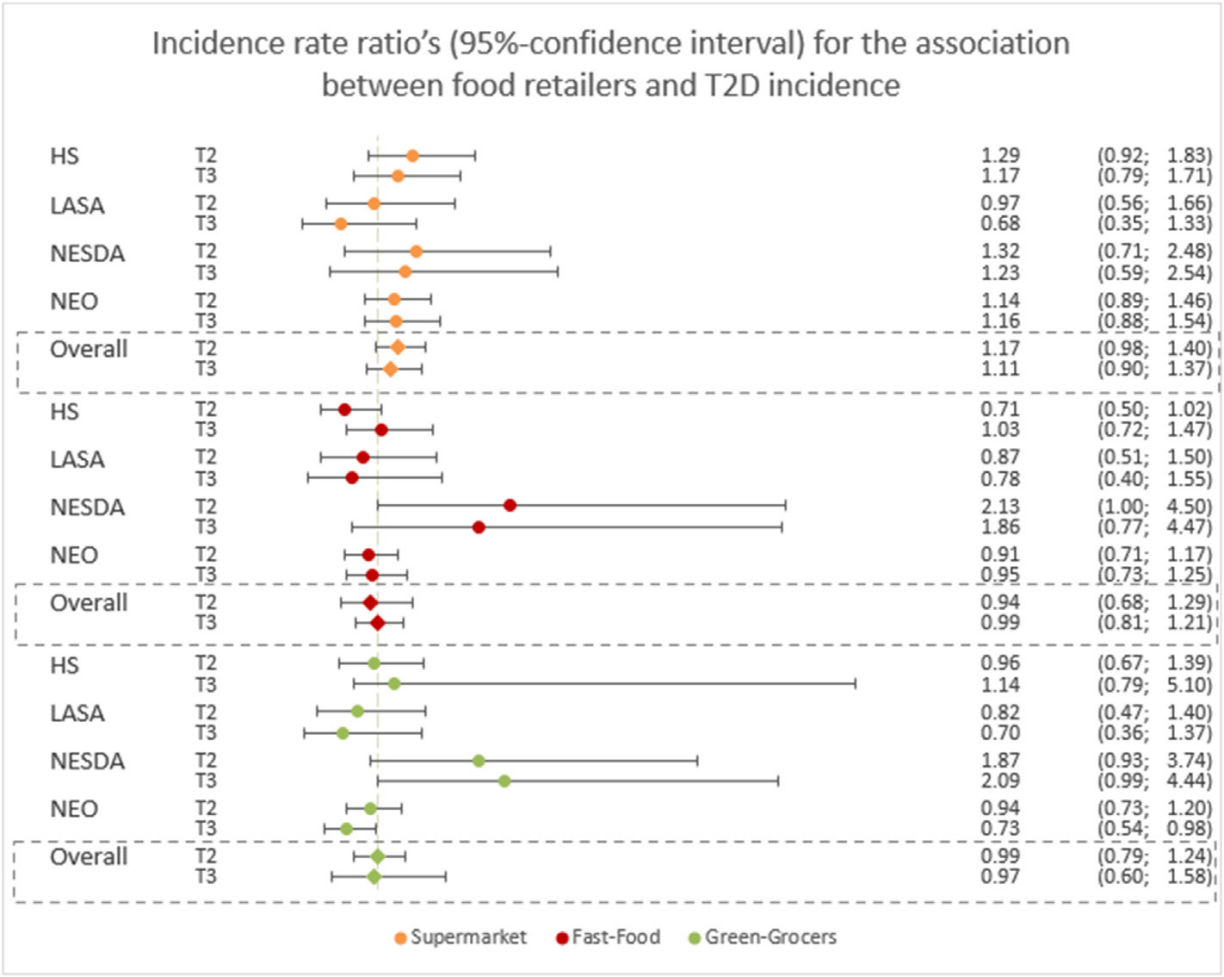
Incidence rate ratio’s (95% confidence interval) for the association between distance to food retailers (tertile comparisons) and type 2 diabetes (T2D) incidence, per cohort and pooled over the cohorts. T2D, type 2 diabetes.

A total of 10,249 participants without T2D at baseline were included in the analyses (range, 899−5893). A flow chart of the study population is presented in Supplemental Figure 1. Of these, 569 developed T2D (HS: *n* = 178; LASA: *n* = 68; NESDA: *n* = 54; NEO: *n* = 269) over a mean follow-up of 6 y (range, 6−10 y). Compared with T1, further distance to supermarkets was associated with higher T2D incidence, although the effect estimates were nonsignificant (T2 vs. T1: IR = 1.17 (0.98, 1.40), T3 vs. T1: IR = 1.11 [0.90, 1.37]) (Figure 2), as well as in the continuous analyses (IR_100m_ = 1.02 [0.99, 1.06]) (Supplemental Figure 2). Associations were similar after minimal adjustment for confounders (Supplemental Table 2; models 1 and 2) and after complete adjustment (Figure 2; model 3). Results across cohorts for supermarkets were homogeneous in all analyses (I^2^ = 0%).

Distance to fast-food outlets was not associated with T2D incidence in the analyses with tertiles (T2 vs. T1: IR = 0.94 (0.68, 1.29), T3 vs. T1: IR = 0.99 [0.81; 1.21]) (Figure 2), as well as in the continuous analyses (IR_100m_= 0.99 [0.96; 1.03]). Overall, associations were homogeneous across cohorts (I^2^ = 0% in T3 vs. T1 and continuous; I^2^ = 55% in T2 vs. T1).

Also, distance to green grocers was not associated with higher T2D incidence in the tertile analyses (T2 vs. T1: IR = 0.99 [0.79, 1.24], I^2^ = 22%, T3 vs. T1: IR = 0.97 [0.60; 1.58], I^2^ = 59%), and in the continuous analyses (IR_100m_ = 1.01 [0.95, 1.07], I^2^ = 47%).

Significant effect modification by age group was found in the association between distance to green grocers and T2D incidence in HS and LASA (Supplemental Table 3). The pattern of effect modification was contradictory in both cohorts, where in HS the association among the youngest age category pointed toward a higher risk with larger distance to green grocers, whereas in LASA the association among youngest age category pointed toward a lower risk with larger distance. In NEO significant effect modification by urbanization level was observed in the association of distance to supermarkets and fast-food with T2D, with only small differences between the strata (IR of 0.96 in urban vs. 0.98 in rural).

Due to a lack of consistency across cohorts, we did not stratify our pooled analyses by these effect modifiers. Stratified analyses per cohort are presented in Supplemental Table 3, only for the continuous distances.

### Mediation analyses

In NEO and HS (pooled *n* = 7549), we did not observe significant mediation by dietary pattern. Mediation analyses revealed associations on the a-path, where each additional 100 m distance from supermarkets or green grocers was associated with 0.1 point (on a scale of 0–13) lower adherence to the DHD15-index (β_supermarket_ = −0.1 [95% CI: −0.3; 0.0], β_green grocer_ = −0.1 [95% CI: −0.1, 0.0]). Also, for fast-food outlets, living 100 m further away was consistently associated with a 0.1-point higher adherence to the DHD15-index (β = 0.1 [95% CI: 0.0, 0.2]). On the b-path, a higher adherence to the DHD15-index was associated with lower T2D incidence (β = −0.07 [95% CI: −0.12, −0.01] or IR = 0.93 [95% CI: 0.89, 0.99]) (Table 4).

**TABLE 4:** Mediation model for the association between distance to supermarkets, fast-food outlets, or green grocers (per 100 m) and type 2 diabetes (T2D) incidence, via adherence to the Dutch Healthy Diet index 15 (DHD15-index); per 10, in HS (*n* = 1635) and NEO (*n* = 5893). Path A represents the association between distance to the food retailer (x) and adherence to the DHD15-index (m). Path B represents the association between adherence to the DHD15-index (m) and T2D incidence (y). Path C’ represent that main association (x–y), additionally adjusted for the mediator (m).

Mediation effect	C’-path (distance to food retailer-> diabetes incidence)		A-path (distance to food retailer -> DHD15-index)		B-path (DHD15-index->T2D incidence)		Indirect effect (a∗b)	
	B	95% CI	B	95% CI	B	95% CI	B	95% CI
Supermarkets								
HS	0.03	−0.04, 0.09	**−0.04**	**−0.07, −0.0**	−0.06	−0.16, 0.05		
NEO	0.02	−0.02, 0.06	−0.01	−0.02, 0.01	**−0.07**	**−0.14, −0.00**		
Pooled	0.02	−0.01, 0.06	**−0.01**	**−0.03, 0.00**	**−0.07**	**−0.12, −0.01**	0.00	0,00, −0.00
Fast food								
HS	0.03	−0.03, 0.08	**0.02**	**0.00, 0.05**				
NEO	−0.01	−0.05, 0.02	**0.01**	**−0.00, 0.02**				
Pooled	0.00	−0.03, 0.03	**0.01**	**0.00, 0.02**	**−0.07**	**−0.12, −0.01**	−0.00	−0.00, −0.00
Green grocers								
HS	0.02	−0.01, 0.05	**0.01**	**0.00, 0.02**				
NEO	−0.03	−0.05, −0.01	**−0.01**	**−0.01, 0.00**				
Pooled	−0.02	−0.03, 0.00	**−0.01**	**−0.01, −0.00**	**−0.07**	**−0.12, −0.01**	0.00	0.00, 0.00

Models are fully adjusted (model 3).

*Abbreviations:* DHD15-index, Dutch Healthy Diet index 2015; HS, Hoorn Studies; NEO, the Netherlands Epidemiology of Obesity; T2D, type 2 diabetes; CI, confidence interval.

### Pooled analyses—sensitivity analyses

Sensitivity analyses using the presence of food retailers as the main determinant of T2D incidence also did not result in significant associations. The direction of association was similar to that of the main analyses. A further distance to supermarkets was pointing to a higher risk in the main analyses, and the presence of a supermarket in 400-m buffer was pointing toward a lower risk (β = 0.91 [95% CI: 0.73, 1.12]). The presence of a fast-food outlet pointed toward higher risk (β = 1.08 [95% CI: 0.79, 1.49]), in line with the direction in the main analyses. For green grocers only very weak associations were observed (Supplemental Figure 3).

With regard to individual food group analyses, we observed that higher distance to fast-food outlets was associated with higher adherence to the fruit intake recommendations (Supplemental Table 4). Overall, associations were very small and not consistent over both cohorts.

## Discussion

In this prospective multicohort study, we investigated whether distance to food retailers in the residential neighborhood was associated with T2D incidence. Furthermore, we examined the mediating role of diet quality in this association. This study did not show an association between spatial accessibility of food retailers and T2D incidence. Nevertheless, mediation analyses revealed small associations consistent with the hypothesized pathway. Residents living further away from supermarkets and green grocers had poorer diet quality and those further away from fast-food outlets had better diet quality. A better diet quality, in turn, was associated with a reduced T2D risk.

Recent systematic and narrative reviews concluded that the association between RFE and T2D were mostly cross-sectional in design, and yielded mixed results [5,[7], [8], [9]]. In line with earlier prospective studies, the present study confirmed that there was no association between accessibility to food retailers and T2D incidence, although the association of distance to supermarket with T2D was in the expected (positive) direction [[15], [16], [17], [18]]. T2D is a distal outcome from exposure to food retailers to development of disease. Therefore, an argument for mediation analyses is to break down this distal pathway, to gain insight into the proposed pathway. In our mediation analyses, we indeed observed subtle associations on the mediating paths that were in line with the proposed pathway that we expected. Further distance to supermarkets or green grocers was associated with a poorer diet quality and further distance to fast-food outlets was associated with better diet quality. A significant direct association was however not identifiable, potentially owing to the small effects down this distal pathway. The mediation analyses, therefore, proves to be a valuable addition to gain insights and unravel the putative pathway toward the development of T2D, even if the main association is nonsignificant [39]. Compared with earlier studies, this is the first to assess all these measures in a single comprehensive study and to apply a formal mediation analysis.

Two previous prospective studies from Sweden and Canada observed associations between higher exposure to fast-food outlets and higher T2D incidence [[17], [18], [19]]. We observed associations in a similar direction, which did not reach statistical significance. A difference was that these studies used a density measure of fast-food outlets to total food retailers, in a 720-m and 1000-m Euclidean buffer, or in administrative neighborhood areas [[19], [20], [21]]. This association was investigated in a Dutch cross-sectional study and such a ratio was not associated with T2D [12]. Previous Dutch studies did show increasingly stronger associations with presence of fast-food outlet, especially using smaller buffers (100–500 m) [12,13]. Therefore, we investigated a similar exposure measure to these Dutch studies in our sensitivity analyses, and although the directions of the associations observed in our study were intuitive and in line with the main analyses, these did not reach statistical significance. In these earlier Dutch studies, associations were statistically significant, but only of a very small magnitude, indicating that a very large sample size is required to detect a statistically significant association. Although effect sizes are small on individual level, all individuals are to some degree exposed creating a potentially large public health impact on population level.

Moreover, the mediation analyses showed that shorter distances to supermarkets and green grocers and larger distance to fast-food outlets were associated with a better diet quality. Also, better diet quality was associated with a lower T2D incidence, in line with our previous work [10]. We found that associations were small and we may not have had the statistical power (especially regarding the outcome T2D incident cases) to reach statistical significance. The importance of statistical power is illustrated in earlier studies, where a single study observed small but significant differences between highest and lowest quartile of fast-food density and change in fasting plasma glucose (0.8 mg/dL) in 4010 participants [14]. In the same study, however, no association was found between fast-food density and T2D incidence, and 4.3% developed T2D. Whereas in our study, 2 cohorts had an incidence of 3% (NEO) and 1% (NESDA). In the UK Biobank, a cross-sectional analysis indicated highest distance to hot food take outs (>1500 m) was significantly associated with 16% lower odds of having diabetes, where the sample consisted of ∼350,000 participants and 3% had T2D [42]. This comparison between studies illustrates the importance of high validity T2D measurements and a very large sample size. However, the effect size in the UK study was also larger, indicating that for instance a larger variation in exposures in the UK setting (mean distance of 1886 m [SD = 2177 m] compared with 516.9 m [SD = 466.7 m] in our study) contributed to these results. Also, measuring specific dietary behaviors, such as fast-food consumption, could be of added value. Unfortunately, the FFQ in these studies did not assess these food groups specifically.

The heterogeneity of the associations between food retailers and T2D incidence across cohorts in the current pooled analysis was generally low, especially in the analyses with continuous outcomes. However, slight nuances were seen in the continuous analyses, which indicates the importance of using standardized measures across studies to better enable comparison between these studies. Also, some evidence for effect modification by age, sex, education, and urbanity was observed within cohorts in the continuous analyses, however, these were inconsistent and did not show clear patterns.

Limitations of the present study should be noted. First, we investigated the RFE, although of course one is exposed to more than just the residential environment. An earlier study suggested that, the work and commute environment are also important to explain associations between exposure to food outlets and obesity [43]. Second, the RFE is subject to change, and in a recent study, we observed that the “foodscape” in the Netherlands was characterized by an increase in food delivery and a decrease in specialty shops [44]. Studies up until now, including ours, have not investigated associations between changes in food environment to changes in T2D incidence or how long food establishments may have been in their present, although this may be of added value. One study relating a food environment healthiness index over time to cardiovascular disease (CVD) in the Netherlands found that an increased exposure to healthier food environments was associated with reduced risk of CVD [45]. Moreover, food environments could change because people moved residence, but we could not verify how long people had been living in this location in all cohorts, although we expect these populations to be relatively stable over time in their place of residence. Also, we could not take the use of food delivery places into account in our cohorts, but this might have attenuated our associations, as participants’ source of fast food could be mainly delivered to the home, rather than using the fast-food outlet that is closest to home. Third, due to the choice of using high-quality, national, and harmonized exposure data, we had to accept concessions in the quality of outcome assessment, as outcomes were measured differently across the different cohorts. However, we did adhere to the WHO criteria of diabetes incidence. Fourth, the study may have been underpowered, mainly due to the relatively low overall number of incident T2D cases. Fifth, it may have been interesting to investigate changes in BMI, as an alternative outcome, however, we did not have this data in all cohorts. Finally, spatial accessibility captures only part of the food environment picture, because in-store environments are diverse. Especially supermarkets have a heterogeneous retail offer, and therefore future studies should include data on in-store food environment, individual preferences, and individuals’ interactions with stores, to gain a more complete insight into how RFE can be associated with risk of T2D or other chronic diseases [46].

The study also had several strengths. We used prospective cohort data, collected across different regions in the Netherlands, and used a homogeneous measure of exposure assessment to standardize across cohorts. Also, the use of multiple cohorts provided increased variation in the exposure to the RFE. In addition, this study design enabled us to investigate mediating pathways and unravel the putative pathways on the distal association from RFE to T2D. Finally, we were able to use a valid and standardized objective data source for assessing exposure to RFE across the Netherlands, as opposed to self-reported measures that were often used in the literature.

In conclusion, this study did not provide evidence for an association between RFE and T2D incidence. We did observe small associations of shorter distances to supermarkets and green grocers, and larger distances to fast-food outlets with better diet quality. A higher diet quality, in turn, was associated with a lower risk of T2D. In terms of implications for practice, this study adds some evidence to support a call for healthier food environments in the Netherlands. Given the changes in RFE and heterogeneity of retail offer within stores, future research should focus on longitudinal exposures and how individuals interact with their food environment.

## Author contributions

NdB, JL, FR, JBJWJB designed research; NdB conducted research; BWJHP, EG, MV, EJT, RdM EWVE provided essential materials; NdB analyzed data and wrote paper; JWBJB had primary responsibility for final content. All authors contributed to the revision and editing of the manuscript; and all authors: read and approved the final manuscript.

## Data availability

The data described in the manuscript will not be made publicly available. Further information, including the procedures for obtaining and accessing data from GECCO and the included cohort studies, is described at: https://www.gecco.nl.

## Funding

NR den Braver reports that financial support was provided by the Amsterdam University Medical Centers. The study was supported by EXPOSOME-NL, which is funded through the Gravitation program of the Dutch Ministry of Education, Culture, and Science and the Netherlands Organization for Scientific Research (NWO grant number 024.004).

## Conflict of interest

The authors declare that they have no known competing financial interests or personal relationships that could have appeared to influence the work reported in this paper.
