# Supplementary material for: Neighborhood Retail Food Environment, Diet Quality and Type 2 Diabetes Incidence in 4 Dutch Cohorts
Source: J Nutr. 2025 Apr 30;155(7):2367–75. doi: 10.1016/j.tjnut.2025.04.022 (PMC12308082; doi:10.1016/j.tjnut.2025.04.022)
Supplement: Supplementary file 1 [file mmc1.docx]

# Neighborhood retail food environment, diet quality and type 2 diabetes incidence in four Dutch cohorts

## NR den Braver

Supplementary files

Supplementary File 1: Description cohorts

*Hoorn Study*

The Hoorn Study is a prospective cohort study in the region of Hoorn, first initiated in 1989. For the second wave, participants were recruited from the general population in the city of Hoorn, between 2006 and 2007 (n=2807). Inclusion criteria were living in the Hoorn region, 40-65 years old at time of inclusion and ability to provide informed consent. The follow-up measurement was performed between 2013 and 2015 (n=1734). At baseline a general questionnaire and Food Frequency Questionnaire (FFQ) (32) were administered. At baseline fasting blood samples were drawn to determine fasting plasma glucose levels (FPG), 2-hour glucose (2hG) levels after an 75-g oral glucose tolerance test (OGTT) and HbA1c levels were determined. At follow-up no OGTT was conducted, and HbA1c was measured in a capillary blood sample.

*Longitudinal Aging Study Amsterdam*

The Longitudinal Aging Study Amsterdam is an ongoing, prospective cohort study in the Netherlands on the determinants, trajectories and consequences of physical, cognitive, emotional and social functioning in older adults (27, 28, 47). Sampling, response and procedures are described in detail elsewhere (27). In summary, a random sample of older men and women (55-85 years), stratified by age and sex, was drawn from the population registries of eleven municipalities across three culturally distinct regions (Amsterdam, Zwolle, Oss) in the Netherlands, thereby representing variation in religious background and urbanity. The baseline data collection was conducted in 1992/93 and the baseline sample included 3,107 respondents. Since then, follow-up measurements have been conducted approximately every three years. Additional respondents aged 55-64 years were recruited from the same sampling frame in 2002/03. For the present study we used data from wave 2005/06 as baseline, and data from wave 2015/16 as follow-up. In total, 2,165 respondents were included at wave 2005/06, of which 1171 also participated at wave 2015/16. Both baseline and follow-up diabetes was based on diagnosis by general practitioners, medication use, or self-report.

*The Netherlands Epidemiology of Obesity*

The Netherlands Epidemiology of Obesity (NEO) study is a population-based prospective cohort in 6671 middle-aged men and women, designed to investigate pathways that lead to obesity-related diseases. Between 2008 and 2012 participants were recruited from the general population in the area of Leiden in the West of the Netherlands. Inclusion criteria were 45-65 years old at time of inclusion, and participants with a self-reported BMI≥27 were oversampled. At baseline a general questionnaire and FFQ (32) were administered, and a fasting blood sample was drawn. Diabetes was based on general practitioners’ diagnoses at baseline, and during a mean follow-up of 7 years (n=5,893).

*The Netherlands Study of Depression and Anxiety*

Participants were recruited between 2004-2007 from the general population, primary care and specialized mental health care facilities, to include persons with or without current or remitted depressive or anxiety disorders (n=2981). Inclusion criteria were age 18-65 years old, and exclusion criteria were a diagnosis of a psychotic disorder, obsessive compulsive disorder, bipolar disorder, or severe addiction and not being fluent in Dutch. For the present study, we used measurement wave 5 as follow-up, performed in 2010-2013 (n=2,256). At baseline and follow-up, a general questionnaire was administered and fasting blood samples were drawn. Diabetes was based on FPG, self-report or medication use.

Supplementary Table 1: calculation of DHD15-index

| **Component** | | **Dutch dietary guidelines 2015** | **Minimum score = 0 (threshold level)** | **Maximum score = 10**  **(cut-off level)** |
| --- | --- | --- | --- | --- |
| *Adequacy components** | | | | |
| 1. | Vegetables | Eat at least 200 grams of vegetables a day. | 0 g/day | ≥ 200 g/day |
| 2. | Fruit | Eat at least 200 grams of fruit a day. | 0 g/day | ≥ 200 g/day |
| 3. | Wholegrain products | Eat at least 90 g of wholegrain products daily | 0 g/day | ≥ 90 g/day |
| 4. | Nuts | Eat at least 15 grams of unsalted nuts a day. | 0 g/day | ≥ 15 g/day |
| 5. | Legumes | Eat legumes each week. | 0 g/day | ≥ 10 g/day |
| 6. | Fatty fish | Eat one time fatty fish each week. | 0 g/day | ≥ 15 g/day |
| 7. | Tea | Drink three cups of tea a day. | 0 ml/day | ≥ 450 ml/day |
| *Moderation components** | | | | |
| 8. | Red meat | Limit the consumption of red meat. | ≥ 100 g/day | ≤ 45 g/day |
| 9. | Processed meat | Limit the consumption of processed meat. | ≥ 50 g/day | 0 g/day |
| 10. | SSBs  juices | Limit the consumption of SSBs and fruit juices. | ≥ 250 ml/day | 0 ml/day |
| 11. | Alcohol | Limit the consumption of alcohol each day. | Women ≥ 20 g/day (ethanol) Men ≥ 30 g/day (ethanol) | Women ≤ 10 g/day (ethanol) Men ≤ 10 g/day (ethanol) |
| *Optimum component** | | | | |
| 12. | Dairy products | Take a few portions of dairy products each day, including yoghurt and milk. | 0 g/day OR ≥ 750 g/day | 300 - 450 g/day |
| *Ratio component** | | | | |
| 13. | Fats and oils | Replace hard margarine, hard cooking fats, and butter (hard fats/oils) by soft margarines, liquid cooking fats, and vegetable oils (soft fats/oils). | No intake of soft fats and oils OR  Ratio soft fats and oils/solid fats ≤ 0.7 | No intake of solid fats OR ratio soft fats and oils/solid fats ≥ 13 |
| 3. | Wholegrain products | Replace refined cereal products by wholegrain products | No intake of wholegrain products OR Ratio wholegrain/refined grain ≤ 0.6 | No intake of refined products OR ratio wholegrain/refined grain ≥ 11 |

SSB = sugar sweetened beverage,*calculation of components: adequacy score= $\frac{intake}{cut-off value}*10$, moderation score=$10-\frac{intake}{threshold value}*10$, optimum score = $10- \frac{10*(intake-cut-off value)}{optimum range}*10$, ratio component = $10* \frac{ratio of intake-threshold value}{ratio range}$

Supplementary Table 2: Incidence ratio’s (95%CI) for the association between distance to food retailers and T2D incidence, per cohort and pooled, adjustment models 1 and 2.

|  | Tertiles of distance | | |  |
| --- | --- | --- | --- | --- |
|  | **T1** | **T2** | **T3** | **Continuous (per 100 m)** |
| Supermarket |  |  |  |  |
| HS (178/1656 cases) |  |  |  |  |
| Model 1 | 1 | 1.32 (0.93; 1.86) | 1.28 (0.90; 1.82) | 1.04 (0.96; 1.12) |
| Model 2 | 1 | 1.33 (0.95; 1.88) | 1.31 (0.92; 1.85) | **1.09 (1.00; 1.19)** |
| Model 3 |  |  |  |  |
| lasa (68/899 cases) |  |  |  |  |
| Model 1 | 1 | 0.96 (0.55; 1.66) | 0.63 (0.34; 1.16) | 0.86 (0.69; 1.09) |
| Model 2 | 1 | 0.95 (0.56; 1.60) | 0.63 (0.35; 1.14) | 0.87 (0.70; 1.10) |
| Model 3 |  |  |  |  |
| NESDA (54/1801 cases) |  |  |  |  |
| Model 1 | 1 | 1.31 (0.70; 2.45) | 1.22 (0.64; 2.32) | 0.96 (0.75; 1.23) |
| Model 2 | 1 | 1.32 (0.71; 2.44) | 1.21 (0.63; 2.35) | 0.96 (0.74; 1.24) |
| Model 3 |  |  |  |  |
| NEO (269/5593) |  |  |  |  |
| Model 1 | 1 | 1.09 (0.85; 1.39) | 1.05 (0.82; 1.36) | 0.99 (0.96; 1.02) |
| Model 2 | 1 | 1.05 (0.82; 1.35) | 1.07 (0.83; 1.38) | 0.99 (0.96;1.02) |
| Model 3 | 1 | 1.14 (0.89; 1.46) | 1.16 (0.88; 1.54) | 1.02 (0.98; 1.07) |
| Pooled |  |  |  |  |
| Model 1 | 1 | 1.20 (0.94; 1.54) | 1.10 (0.80; 1.51) |  |
| Model 2 | 1 |  |  |  |
| Model 3 |  |  |  |  |
| Fast-food |  |  |  |  |
| HS |  |  |  |  |
| Model 1 | 1 | 0.73 (0.51; 1.05) | 1.12 (0.81; 1.56) | 1.06 (0.98; 1.17) |
| Model 2 | 1 | 0.74 (0.52; 1.05) | 1.14 (0.83; 1.58) | 1.08 (0.98; 1.18) |
|  |  |  |  |  |
| lasa |  |  |  |  |
| Model 1 | 1 | 0.83 (0.49; 1.41) | 0.66 (0.37; 1.16) | 0.87 (0.70; 1.10) |
| Model 2 | 1 | 0.84 (0.50; 1.43) | 0.68 (0.38; 1.20) | 0.88 (0.71; 1.10) |
|  |  |  |  |  |
| NESDA |  |  |  |  |
| Model 1 | 1 | 1.94 (0.96; 3.93) | 1.69 (0.81; 3.53) | 1.01 (0.79; 1.29) |
| Model 2 | 1 | 2.05 (1.00; 4.20) | 1.73 (0.81; 3.67) | 1.01 (0.79; 1.30) |
|  |  |  |  |  |
| NEO |  |  |  |  |
| Model 1 | 1 | 0.90 (0.70; 1.14) | 0.85 (0.66; 1.09) | 0.97 (0.94; 1.00) |
| Model 2 | 1 | 0.88 (0.69; 1.12) | 0.85 (0.66; 1.10) | 0.97 (0.94; 1.00) |
| Model 3 |  | 0.91 (0.71; 1.17) | 0.95 (0.72; 1.25) | 0.99 (0.95; 1.02) |
| Pooled |  |  |  |  |
| Model 1 | 1 | 1.01 (0.68; 1.51) | 1.09 (0.78; 1.52) |  |
| Model 2 | 1 |  |  |  |
| Green grocers |  |  |  |  |
| HS |  |  |  |  |
| Model 1 | 1 | 1.09 (0.77; 1.55) | 1.23 (0.88; 1.72) | 1.05 (0.97; 1.13) |
| Model 2 | 1 | 1.08 (0.76; 1.53) | 1.24 (0.88; 1.73) | 1.05 (0.97; 1.13) |
|  |  |  |  |  |
| lasa |  |  |  |  |
| Model 1 | 1 | 0.81 (0.47; 1.39) | 0.61 (0.34; 1.08) | 0.98 (0.83; 1.15) |
| Model 2 | 1 | 0.80 (0.47; 1.37) | 0.62 (0.35; 1.10) | 0.98 (0.83; 1.15) |
|  |  |  |  |  |
| NESDA |  |  |  |  |
| Model 1 | 1 | 1.78 (0.89; 3.56) | 1.88 (0.92; 3.82) | 1.10 (0.92; 1.31) |
| Model 2 | 1 | 1.80 (0.90; 3.61) | 1.84 (0.90; 3.77) | 1.09 (0.90; 1.30) |
|  |  |  |  |  |
| NEO |  |  |  |  |
| Model 1 | 1 | 0.93 (0.73; 1.17) | 0.70 (0.54; 0.91) | **0.97 (0.95; 0.99)** |
| Model 2 | 1 | 0.93 (0.73; 1.18) | 0.71 (0.54; 0.92) | **0.97 (0.95 ; 0.99)** |
| Model 3 |  | 0.94 (0.73; 1.20) | 0.73 (0.54; 0.98) | 0.97 (0.95; 0.99) |
|  |  |  |  |  |
| Pooled |  |  |  |  |
| Model 1 | 1 | 1.13 (0.87; 1.49) | 1.18 (0.77; 1.80) |  |
| Model 2 | 1 |  |  |  |

HS = Hoorn Study, LASA = Longitudinal Aging Study Amsterdam, NESDA = The Netherlands Study of Depression and Anxiety, NEO = The Netherlands Epidemiology of Obesity. Model 1: Adjusted for age, sex, education, and follow-up duration, Model 2: additionally, for physical activity and smoking, ethnicity.

Supplementary Table 3: interactions within cohorts

Supplement 3.1: Incidence rate ratio’s (95%CI) for the association between distance to green grocers and T2D incidence, stratified by **age** in **HS** (interaction p=<0.01)

| HS | Distance (per 100 m) |
| --- | --- |
| Green GRocers |  |
| Model 3 (<54 year) | 1.11 (0.97; 1.26) |
| Model 3 (>54 year) | 1.00 (0.91; 1.10) |
| *Fully adjusted models adjusted for demographics, lifestyle and broader RFE | |

Supplement 3.2: Incidence rate ratio’s (95%CI) for the association between distance to green grocers and T2D incidence, stratified by **age** in **LASA** (interaction p = <0.01)

| LASA | Distance (per 100 m) |
| --- | --- |
| Green GRocers |  |
| Model 3 (<72 years) | 0.83 (0.65; 1.07) |
| Model 3 (>72 years) | 1.16 (0.99; 1.36) |
| *Fully adjusted models adjusted for demographics, lifestyle and broader RFE | |

Supplement 3.3: Incidence rate ratio’s (95%CI) for the association between distance to supermarket and T2D incidence, stratified by **urbanity** (interaction p=0.01) in **NEO**

| NEO | Distance (per 100 m) |
| --- | --- |
| Supermarket - urbanity |  |
| Model 3 (urban) | 0.96 (0.90; 1.02) |
| Model 3 (rural) | 0.98 (0.92; 1.04) |
| *Fully adjusted models adjusted for demographics, lifestyle and broader RFE | |

|  | A path (Distance to food retailer -> DHD15-index)  NHS | | A path (Distance to food retailer -> DHD15-index)  NEO | |
| --- | --- | --- | --- | --- |
|  | *B* | *95%-CI* | *B* | *95%-CI* |
| Supermarkets |  |  |  |  |
| *Vegetables* | -0.009 | -0.089; 0.072 | -0.003 | -0.025; 0.019 |
| *Fruits* | 0.004 | -0.109; 0.118 | -0.0008 | -0.033; 0.031 |
| *Grains* | -0.008 | -0.027; 0.010 | 0.003 | -0.003; 0.009 |
| *Legumes* | -0.033 | -0.178; 0.111 | **-0.057** | **-0.097; -0.017** |
| *Nuts* | 0.050 | -0.066; 0.165 | 0.060 | 0.028; 0.093 |
| *dairy* | -0.015 | -0.109; 0.080 | 0.010 | -0.016; 0.037 |
| *fish* | -0.008 | -0.104; 0.088 | 0.017 | -0.010; 0.044 |
| *tea* | 0.064 | -0.052; 0.180 | 0.015 | -0.016; 0.046 |
| *fat* | 0.084 | -0.038; 0.205 | 0.014 | -0.024; 0.053 |
| *red meat* | -0.049 | -0.118; 0.021 | 0.001 | -0.021; 0.024 |
| *Processed meat* | -0.072 | -0.170; 0.026 | -0.009 | -0.037; 0.019 |
| *SSB* | **-0.159** | **-0.273; -0.045** | -0.013 | -0.045; 0.019 |
| *Alcohol* | 0.018 | -0.108; 0.145 | -0.007 | -0.044; 0.030 |
|  |  |  |  |  |
| Fast-food |  |  |  |  |
| *Vegetables* | -0.015 | -0.093; 0.064 | 0.006 | -0.012; 0.024 |
| *Fruits* | **0.144** | **0.034; 0.254** | **0.030** | **0.002; 0.057** |
| *Grains* | -0.001 | -0.019; 0.017 | 0.005 | -0.000; 0.010 |
| *Legumes* | 0.067 | -0.074; 0.207 | **-0.062** | **-0.096; -0.027** |
| *Nuts* | 0.102 | -0.011; 0.215 | 0.046 | 0.018; 0.074 |
| *dairy* | **0.101** | **0.009; 0.192** | 0.020 | -0.002; 0.043 |
| *fish* | 0.052 | -0.04; 0.146 | 0.008 | -0.015; 0.031 |
| *tea* | 0.085 | -0.028; 0.197 | **0.031** | **0.005; 0.058** |
| *fat* | 0.077 | -0.038; 0.191 | 0.023 | -0.009; 0.056 |
| *red meat* | **-0.069** | **-0.136; -0.002** | 0.010 | -0.010; 0.029 |
| *Processed meat* | **-0.131** | **-0.227; -0.034** | 0.012 | -0.012; 0.036 |
| *SSB* | -0.089 | -0.200; 0.023 | -0.002 | -0.029; 0.025 |
| *Alcohol* | 0.104 | -0.019; 0.227 | 0.014 | -0.017; 0.045 |
|  |  |  |  |  |
| Green Grocers |  |  |  |  |
| *Vegetables* | 0.044 | -0.021; 0.108 | -0.006 | -0.017; 0.005 |
| *Fruits* | 0.084 | -0.007; 0.174 | **-0.023** | **-0.039; -0.007** |
| *Grains* | *-0.006* | *-0.021; 0.009* | *0.001* | *-0.002; 0.004* |
| *Legumes* | -0.050 | -0.166; 0.066 | **-0.029** | **-0.049; -0.009** |
| *Nuts* | 0.057 | -0.035; 0.150 | **0.026** | **0.010; 0.043** |
| *dairy* | 0.070 | -0.006; 0.146 | 0.002 | -0.011; 0.015 |
| *fish* | 0.001 | -0.076; 0.078 | -0.013 | -0.026; 0.001 |
| *tea* | 0.088 | -0.005; 0.180 | 0.011 | -0.005; 0.026 |
| *fat* | -0.015 | -0.113; 0.082 | 0.015 | -0.004; 0.035 |
| *red meat* | 0.007 | -0.048; 0.063 | 0.003 | -0.008; 0.014 |
| *Processed meat* | -0.028 | -0.107; 0.051 | -0.009 | -0.023; 0.005 |
| *SSB* | -0.062 | -0.153; 0.030 | 0.003 | -0.013; 0.019 |
| *Alcohol* | -0.010 | -0.111; 0.091 | 0.004 | -0.015; 0.022 |

Supplementary Table 4: Sensitivity analyses for the association between distance to supermarkets,

fast-food outlets or green grocers (per 100m) and adherence to the DHD15-index individual food groups (score range from 0-10), in NHS (n=1635) an d NEO (n=5893).

Supplementary Figure 1: Flow chart of study population

Analytic population

n = 10,249

Exclusion diabetes at baseline or missing:

N=2,315

Total sample

n = 12,564

Supplementary Figure 2: Regression coefficients for the association between distance (per 100m) to supermarkets, fast-food outlets or green grocers, and T2D incidence, presented as beta (95%-confidence interval) per cohort and pooled over the cohorts.

HS = Hoorn Study, LASA = Longitudinal Aging Study Amsterdam, NESDA = The Netherlands Study of Depression and Anxiety, NEO = The Netherlands Epidemiology of Obesity study

Supplementary Figure 3: sensitivity analyses with density of food retailers

Incidence rate ratio’s for the association between **presence of** food retailers in 400m buffer and T2D incidence A) Presence vs absence of supermarkets B) ) Presence vs absence of fast-food retailers C) ) Presence vs absence of green grocers
